# Clinical burden of *Clostridioides difficile* infection in infective endocarditis: a single-center experience

**DOI:** 10.1007/s11739-025-04085-0

**Published:** 2025-08-11

**Authors:** Lorenzo Bertolino, Augusto Delle Femine, Anna Maria Carolina Peluso, Raffaella Gallo, Rosa Zampino, Fabian Patauner, Roberto Andini, Fabio Luciano, Iolanda Cafarella, Giuseppe Ruocco, Emanuele Durante-Mangoni

**Affiliations:** 1 Department of Precision Medicine, University of Campania ‘L. Vanvitelli’, Naples, Italy; 2 Department of Advanced Medical & Surgical Sciences, University of Campania ‘L. Vanvitelli’, Naples, Italy; 3 Unit of Internal Medicine and Transplants, AORN Ospedali Dei Colli-Monaldi Hospital, Piazzale Ettore Ruggieri, 80131 Naples, Italy; 4 Unit of Microbiology and Virology, AORN Ospedali Dei Colli-Monaldi Hospital, Naples, Italy

**Keywords:** Infective endocarditis, *Clostridioides difficile* infection, Hospital-acquired diarrhea, Outcome

## Abstract

*Clostridioides difficile* infection (CDI) is a leading cause of diarrhea in hospitalized patients and there are no studies addressing its burden in patients with acute infective endocarditis (IE). We aimed to assess the incidence and clinical correlates of CDI occurring during hospitalization for acute bacterial IE and analyzed the prognostic impact of this complication. A single-center retrospective study was conducted including patient hospitalized for acute IE between 2016 and 2024. CDI was defined as acute-onset diarrhea, followed by positive stool test. Analyses were based on the comparison of CDI and non-CDI cases in the subgroup of patients developing diarrhea during hospitalization. We enrolled 370 IE cases with a median age of 65 [53–74] years and most were male (68.1%). 50 (13.4%) developed diarrhea during hospitalization, of which 10 (20%) had a positive stool test and were considered CDI cases. The resultant incidence of CDI in the study period was 17.04 cases for 10,000 patient bed-days. CDI-positive patients showed an independent increased risk of in-hospital mortality (HR 3.34 [1.014–11.058]; *p* = 0.047). CDI incidence in our cohort of patients hospitalized for acute IE was high and associated to a reduced survival, with CDI cases showing a higher in-hospital mortality rate. Our findings underline the need for prevention and rapid detection of CDI in this setting.

**Supplementary Information:**

The online version contains supplementary material available at 10.1007/s11739-025-04085-0.

## Introduction

*Clostridioides difficile* infection (CDI) is a leading cause of diarrhea in hospitalized patients. According to the latest ECDC epidemiological report, 31,731 CDI cases were observed in eight European countries in 2020 [1], and many more remain undiagnosed due to lack of clinical suspicion and/or inadequate laboratory diagnostic capacity [2].

Together with prolonged hospitalization [3], antimicrobial therapy remains the most relevant risk factor for CDI, disrupting the normal gut microbiome and creating a favorable environment for toxigenic strain expansion and development of colitis [4]. Both are inherent clinical features of infective endocarditis (IE).

Several factors impact the risk of CDI, such as the type of antimicrobial administered. For instance, beta-lactam/beta-lactamase inhibitor (BL/BLI) combinations and carbapenems are antibiotic classes associated with CDI, due to their activity against intestinal anaerobes. Moreover, *C. difficile* appears to be intrinsically resistant to third-generation cephalosporins and this may further increase CDI risk [5]. The duration of antibiotic therapy is another major CDI driver. A 14 day course of antibiotic therapy was linked to an increased risk of CDI (27%) when compared to a reference 7 day course (ARR = 1.27, 95%CI: 1.21–1.30) [6]. Other risk factors include advanced age, female sex, and hypoalbuminemia [3, 7].

Patients hospitalized for acute bacterial IE represent a frail population exposed to prolonged courses of intravenous antimicrobials, usually 2–4 weeks for native valve endocarditis (NVE) or ≥ 6 weeks for prosthetic valve endocarditis (PVE) [2]. Moreover, IE patients are often elderly and comorbid, and they are exposed to healthcare-associated infections that may require additional broad-spectrum antimicrobials [8]. All these factors could substantially increase CDI risk in IE. However, to the best of our knowledge, there are no studies focusing on this condition. Accordingly, in this study we aimed to assess the incidence and clinical correlates of CDI occurring during hospitalization for acute bacterial IE and analyze the prognostic impact associated with CDI development.

## Methods

### Study design

This was a single-center, observational, retrospective analysis of data obtained in the context of an ongoing prospective study (PIEPRO—prospective infective endocarditis prognosis study) enrolling consecutive definite acute IE cases (according to ESC 2015 [9] and subsequently to Duke-ISCVID2023 criteria [10]). We enrolled patients admitted to our center (AORN Ospedali dei Colli—Ospedale Monaldi, University of Campania “L. Vanvitelli”—Naples, Italy) between January 2016 and September 2024. CDI cases were defined as acute-onset diarrhea during hospitalization and a positive nucleic acid amplification test (NAAT) or enzyme immunoassay for glutamate dehydrogenase (GDH) and toxins A/B (CD toxins) on stool [11]. In GDH +/CDtox − cases, CDI was always diagnosed based on a positive stool NAAT. Data collection was approved by our local ethics committee (prot. N. AOC/0011110/2020) and all patients provided written informed consent for the anonymous use of their clinical data. Analyses were based on a comparison among CDI-positive and CDI-negative cases in the subgroup of patients developing diarrhea during hospitalization.

### Study end points

The primary exploratory aim of the study was the incidence of CDI during hospitalization for IE. The secondary end point was in-hospital mortality.

### Analyzed variables

Data analyzed in the study included demographic information (age, sex) and co-morbidities (Charlson Comorbidity Index—CCI). The date of admission to our center was used to calculate the length of stay in patients directly admitted to our facility. For patients transferred from other hospitals, the day of first admission was used. All hemato-chemical parameters were obtained on admission by routine biochemical methods used in our hospital central laboratory. These included complete blood count, C-reactive protein (CRP), NT-proBNP, creatinine, and D-dimers. In this study, chronic kidney disease (CKD) was defined as reduction of the estimated glomerular filtration rate (eGFR) below 60 mL/min/1.73 m^2^ according to CKD-EPI equation. The place of infection acquisition was defined as hospital acquired (HA) when symptom onset occurred > 48 h after admission, healthcare associated (HCA) when patients underwent medical interventions within 3 months of IE onset, or community acquired (CA) in the remaining cases. All patients underwent trans-thoracic echocardiogram (TTE), followed by a transesophageal echocardiogram (TEE) where needed. Details on endocarditis sub-type (native, prosthetic, or cardiac implantable electronic device-related) and vegetation location were collected. We also recorded microbial etiology, data on surgical procedures, and in-hospital outcome. In CDI cases, data regarding main antimicrobial therapy administered as treatment of IE and its duration were also collected, together with the type of test used to diagnose CDI.

### Statistical analysis

Numerical variables are presented as median and interquartile range (IQR), while categorical/nominal data are presented as number and percentage. The Mann–Whitney *U* test or Kruskal–Wallis test (for more than two groups) was used to assess statistical significance of the differences between numerical groups of variables, while the Chi-square test was used to compare differences between the categorical variables. We performed a univariable analysis comparing patients developing diarrhea during hospitalization for IE and patients without diarrhea. Subsequently, we compared (in the subgroup of patients with diarrhea) CDI cases and non-CDI cases. CDI incidence was calculated as number of cases per IE patient bed-days (the total number of days a patient stays in a hospital bed while admitted as an inpatient). Independent predictors of in-hospital mortality were assessed using Cox regression analysis and including in the model variables significantly associated with the outcome of interest at univariable analysis. The significance level was set at 5% and all tests were two tailed. All analyses were performed using the statistical software for Windows Statistical Package for Social Sciences v. 22 (SPSS, Inc., Chicago, Illinois, USA).

## Results

**Fig. 1:**
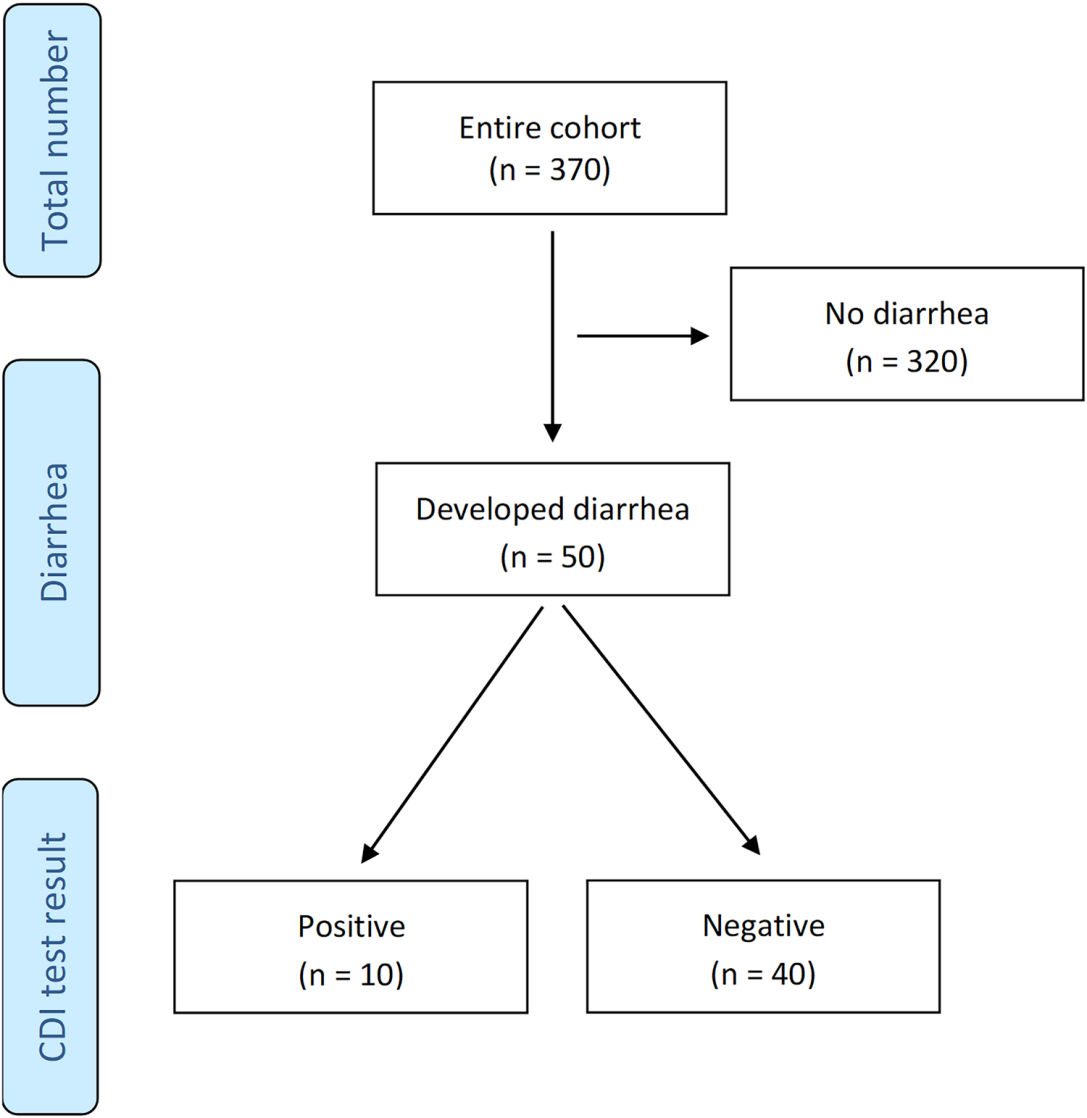
Enrollment flowchart showing the number of cases enrolled in the analyses from the entire cohort

During the study period, 370 IE patients were admitted to our center with acute bacterial IE. Baseline characteristics of the study group are shown in Table 1. Acute diarrhea occurred in 50 patients (13.4%) during antibiotic therapy for IE. Ten patients (20%) were positive and considered CDI cases (Fig. 1). The resultant incidence of CDI in the study period was 17.04 cases for 10,000 patient bed-days. Patients with diarrhea had an increased rate of liver disease and higher inflammatory markers at admission, and experienced a more prolonged hospital stay (33 [24–45] vs 26 [16–39] days; p: 0.008) (Supplementary Table 1). These patients were tested for CDI. NAAT was performed in 24 patients (48%), whereas in 15 patients (30%) the algorithm based on GDH and toxin detection by EIA was adopted. Details of diagnostic tests used are shown in Table 2.

**Table 1:** Baseline clinical features of the 370 IE cases studied

General characteristics	
Total Number	370
Age, years	65 [53–74]
Male gender	252 (68.1)
Female gender	118 (31.9)
Comorbidities	
Chronic kidney disease	128 (34.6)
Ischemic heart disease	84 (22.7)
Chronic heart failure	95 (25.7)
Diabetes mellitus	83 (22.4)
Chronic obstructive pulmonary disease	84 (22.7)
Liver disease	58 (15.7)
Malignant neoplasia	57 (15.4)
Peripheral artery disease	56 (15.1)
Charlson comorbidity index	4 [2–6]
Biochemical data	
NT-proBNP, pg/mL	2551 [708–8189]
Troponin ng/mL	8.7 [0.09—52]
Creatinine, mg/dL	1 [0.8–1.4]
D-Dimers, ng/mL	970 [497–2312]
C-reactive protein, mg/dL	7.6 [3.8–13.7]
White blood cells, mm^3^	10,400 [7700–13930]
Type of acquisition	
Community acquired	270 (73)
Healthcare associated	33 (13)
Hospital acquired	48 (8.9)
Vegetation location	
Aortic valve	132 (35.7)
Mitral valve	105 (28.4)
Multivalve/multisite involvement	61 (16.5)
Lead	38 (10.3)
Tricuspid valve	16 (4.3)
Pulmonary valve	10 (2.7)
Other	8 (2.2)
IE subtype	
Native valve	178 (48.1)
CIED lead	47 (12.7)
Prosthetic biological valve	51 (13.8)
Prosthetic mechanical valve	43 (11.6)
Multi-type involvement	22 (5.9)
Other	17 (4.6)
TAVI	12 (3.2)
Causative microorganism	
* Streptococcus* spp.	86 (23.2)
* Staphylococcus aureus*	76 (20.5)
* Enterococcus* spp.	72 (19.5)
Coagulase-negative staphylococci	52 (14.1)
Gram negatives	7 (1.9)
* Corynebacterium* spp.	4 (1.1)
* Candida* spp.	2 (0.5)
Other microorganisms	4 (1.1)
Culture negative	67 (18.1)
Vegetation size, mm	13.5 [9–20]
Cardiac surgery	
Yes	225 (60.8)
No	145 (39.2)
Length of hospitalization, days	27 [17–40]
In-hospital outcome	
Survivors	300 (81.1)
Non-survivors	70 (18.9)

Categorical variables are presented as number and percentage

Numerical variables are presented as median and IQR

*IE* Infective endocarditis, *NT-proBNP* N-terminal prohormone brain natriuretic peptide

**Table 2:** Details of antimicrobial therapy and CDI diagnostics

Total number	370
Antimicrobial therapy	
Daptomycin	100 (27)
Ampicillin–sulbactam	82 (22.2)
Amoxicillin–clavulanic acid	58 (15.7)
Ceftriaxone	48 (13)
Gentamicin	23 (6.2)
Cefazolin	20 (5.4)
Teicoplanin	15 (4.1)
Vancomycin	8 (2.2)
Linezolid	2 (0.5)
Duration of antibiotic therapy, days	30 [20–42]
Antimicrobials before blood cultures	90 (24.3)
*Clostridioides difficile* testing	
Tested	50 (13.5)
Not tested	320 (86.5)
CDI test result	
Positive	10 (20)
Negative	40 (80)
Type of CDI test	
NAAT	24 (48)
GDH + toxin assay	15 (30)
Culture	4 (8)
Culture and NAAT	4 (8)
NAAT + GDH + toxin assay	3 (6)

Categorical variables are presented as number and percentage

Numerical variables are presented as median and IQR

CDI *Clostridioides difficile* infection, *NAAT* Nucleic acid amplification test, *GDH* Glutamate dehydrogenase

### Univariable analysis comparing CDI cases and non-CDI cases

In the overall cohort, the development of CDI was associated with a significant increase of in-hospital mortality (50% vs 18%; *p* = 0.025).

Table 3 shows results of the univariable analysis comparing CDI and non-CDI cases in the subgroup of patients developing diarrhea during hospitalization. Among the CDI group, the mean age was 64 [55–77.5] years and most patients were females (70%) without significant differences with the non-CDI group. CDI was more likely to occur among IE patients with HCA-IE (40% vs 10%; *p* = 0.002).

**Table 3:** Univariable analyses of clinical, biochemical and outcome variables between the CDI and non-CDI groups (*n* = 50)

Parameter	Univariable analysis		
			*p*-value
	CDI (*n* = 10)	Non-CDI (*n* = 40)	
General characteristics			
Age	64 [55–77.5]	67 [55–73.7]	0.961
Sex			0.157
Male	3 (30)	22 (55)	
Female	7 (70)	18 (45)	
Comorbidities			
CHF (prior to IE onset)	5 (50)	12 (30)	0.232
Ischemic heart disease	4 (40)	9 (22.5)	0.259
Chronic obstructive pulmonary disease	2 (20)	12 (30)	0.529
Diabetes	3 (30)	11 (27.5)	0.875
Liver disease	4 (40)	13 (32.5)	0.654
Malignant neoplasia	1 (10)	7 (17.5)	0.563
Chronic kidney disease	3 (30)	17 (34)	0.470
Peripheral artery disease	2 (20)	7 (17.5)	0.854
Charlson comorbidity index	5 [1-6]	4 [2-6]	0.922
Vegetation size, mm	11.5 [7.7–19.5]	14.5 [7-18]	0.785
Type of acquisition			0.002
Community acquired	5 (50)	27 (54)	
Healthcare associated	4 (40)	1 (2)	
Hospital acquired	1 (10)	11 (22)	
Type of valve			0.100
Prosthetic biological valve	2 (20)	7 (17.5)	
Prosthetic mechanical valve	1 (10)	7 (17.5)	
Native valve	4 (40)	16 (40)	
CIED	0 (0)	6 (15)	
TAVI	2 (20)	0 (0)	
Multisite location	1 (10)	2 (5)	
Other	0 (0)	2 (5)	
Vegetation location			0.314
Aortic valve	4 (40)	10 (25)	
Mitral valve	4 (40)	14 (35)	
Multivalve/multisite involvement	1 (10)	9 (22.5)	
CIED lead	0 (0)	4 (10)	
Tricuspid valve	0 (0)	2 (5)	
Other	0 (0)	1 (2.5)	
Pulmonary valve	1 (10)	0 (0)	
Causative microorganism			0.025
* Streptococcus* spp.	0 (0)	4 (10)	
* Staphylococcus aureus*	1 (10)	13 (32.5)	
Coagulase-negative staphylococci	2 (20)	3 (7.5)	
* Enterococcus* spp.	1 (10)	13 (32.5)	
Gram negatives	1 (10)	1 (2.5)	
* Candida* spp.	0 (0)	1 (2.5)	
* Corynebacterium* spp.	2 (20)	0 (0)	
Negative cultures	3 (30)	5 (12.5)	
Biochemical data, median			
C-reactive protein, mg/dL	7.8 [9.4–23.4]	9.1 [4.5–16.3]	0.042
Creatinine, mg/dL	0.9 [0.4–3.4]	1.05 [0.8–1.6]	0.689
NT-proBNP, pg/mL	5509 [4723–36169]	3364 [1419–5429]	0.033
Troponin ng/mL	23.3 [5–1925]	13.2 [0.08–51.9]	0.145
D-dimers, ng/mL	1769 [826–2312]	1118 [467–2706]	0.789
White blood cells, n/mcL	10,050 [5482–18932]	11,595 [8095–15635]	0.482
Antibiotic therapy			
Amoxicillin–clavulanic acid	1 (10)	5 (12.5)	0.828
Ampicillin–sulbactam	1 (10)	11 (27.5)	0.246
Cefazolin	2 (20)	3 (7.5)	0.239
Ceftriaxone	0 (0)	5 (12.5)	0.239
Daptomycin	3 (30)	10 (25)	0.747
Teicoplanin	0 (0)	1 (2.5)	0.614
Vancomycin	1 (10)	1 (2.5)	0.279
Antimicrobials before blood culture	2 (20)	10 (20)	0.741
Duration of antibiotic therapy, days	29 [19–53.7]	33 [21.2–44.7]	0.627
Length of hospitalization, days	34.5 [13–52.2]	33 [25.7–45.7]	0.780
Cardiac surgery			0.470
Yes	5 (50)	15 (37.5)	
No	5 (50)	25 (62.5)	
In-hospital outcome			0.017
Survivors	5 (50)	34 (85)	
Non-survivors	5 (50)	6 (15)	

Categorical variables are presented as number and percentage

Numerical variables are presented as median and IQR

*CDI Clostridioides difficile* infection, *CHF* Chronic heart failure, *IE* Infective endocarditis, *CIED* Cardiac implantable electronic device, *TAVI* Transcatheter aortic valve implantation, *NT-proBNP* N-terminal prohormone brain natriuretic peptide

Among CDI patients, there were higher rates of native valve involvement (40%), without significant differences in terms of valve location. Negative cultures or uncommon etiologies were more frequently observed in CDI cases. The most common isolated pathogens in non-CDI cases were *Staphylococcus aureus* and *Enterococcus* spp*.* (32.5% each), whereas negative cultures accounted for 30% of the CDI group compared with 12.5% of the non-CDI group.

Regarding antibiotic treatment, the most common antimicrobial administered in CDI was daptomycin (30%), whereas two (20%) patients received cefazolin. No significant differences were found in terms of antimicrobials administered before blood cultures. In non-CDI, the main antibiotics used were ampicillin–sulbactam and daptomycin. We did not find differences between groups in terms of length of hospitalization (34.5 [13–52.2] vs 33 [25.7–45.7]; *p* = 0.780) and duration of antibiotic therapy, it was 29 [19–53.7] days for CDI subjects and 33 [25.7–45.7] days in the non-CDI group (*p* = 0.627). Cardiac surgery was performed during hospitalization in a higher number, albeit not significant, of CDI patients (50% vs 37.5%). In-hospital mortality was higher in CDI patients (50% vs 15%; *p* = 0.017).

### Impact of CDI on in-hospital mortality in patients with diarrhea

At univariable analysis, we confirmed a statistically significant association between the occurrence of CDI during hospitalization for IE and in-hospital mortality. No other significant associations with in-hospital mortality were found (data not shown). A Cox regression analysis found that a CDI-positive status was independently associated with an increased risk of death during IE hospitalization (HR 3.34[1.014–11.058]; *p* = 0.047).

## Discussion

The results of our study show that the prevalence of diarrhea in patients hospitalized for acute IE is similar when compared to the general inpatient population (13.4% vs 12%) [12]. CDI was the cause of diarrhea in a proportion of them (20%), suggesting that other factors also have an impact on diarrhea occurrence in this setting (antimicrobials adverse effects, administration of laxatives, etc.). Indeed, CDI incidence (about 17 cases for 10,000 patient bed-days) was higher than the average observed in previous studies on an unselected hospital population [13]. This is likely due to the presence of several risk factors that identify IE patients as being at high risk for developing this complication. It is interesting to observe that CDI incidence data in our IE cohort was high, despite the relatively low incidence shown in a regional epidemiological report [13]. Indeed, our cohort presented several features that, according to current literature, are listed as major risk factors for CDI. One of the most relevant is the prolonged antimicrobial treatment that in our cohort had a median duration of 30 [20–42] days. In addition, the median age of our study cohort was 65, and older patients (> 65 years) can be considered to be at higher risk for CDI. This may be linked to age-associated modifications in the intestinal microbiota composition [4] as well as immune system aging. Moreover, patients who developed CDI are significantly frail and have an overall high rate of comorbidities, with high prevalence of chronic heart failure and ischemic heart disease, followed by liver disease and chronic kidney disease, as indeed occurred in our cohort [15].

CDI-positive patients showed a significantly increased and independent mortality risk during hospitalization of IE. This result is in line with other studies suggesting a negative impact of CDI on the overall outcome [17]. Moreover, it also applies when analyzing outcomes of patients undergoing cardiac surgery for other indications. Indeed, a study performed in a large cohort of patients undergoing cardiac surgery confirmed the significant association between CDI and in-hospital mortality [18]. Interestingly, in this study CDI was more likely complicating the in-hospital course of patients developing endocarditis after cardiac surgery (OR 4.7; 95% CI 3.91–5.72).

When looking at the comparative analysis between CDI and non-CDI, CDI cases were more likely to show atypical isolations or undemonstrated etiology. By contrast, in our cohort, most patients with staphylococcal and enterococcal IE, well known for an aggressive presentation, showed a lower incidence of CDI. A possible explanation for this figure relies on the need for broader-spectrum antimicrobial therapy in unclear or unknown etiology, which might have had a greater impact on intestinal microbiota and increased the risk for CDI. Moreover, patients with CDI also showed a higher rate of HCA-IE. This might have also played a role in affecting the IE etiology in this subgroup of patients in whom previous HC contacts might have increased administration of other antimicrobial agents or increased the risk for bacteremia due to non-IE typical microorganisms. Therefore, CDI should be more carefully sought in patients with HCA-IE. Of note, HCA-IE has significantly increased recently and occurred more frequently in immunocompromised hosts, in whom CDI may have a higher impact on in-hospital outcome [9, 16].

Among the CDI group, the proportion of patients who underwent surgery was higher, as compared to non-CDI. The reason behind this finding remains unclear. In contrast, CDI development was not associated with increased duration of antibiotic therapy, neither in hospital nor before admission.

Our study has several limitations. First, despite the efforts done to collect data, the retrospective nature of the study poses a risk for underreporting bias. Detailed data regarding etiology of diarrhea in the non-CDI group were not collected. Moreover, the low number of patients included in the univariable analysis may influence the statistical power of our results.

In conclusion, we found a high incidence of CDI in our cohort of patients hospitalized for acute IE, underlying the need for further studies addressing this topic. CDI can have a negative impact on IE outcome by increasing in-hospital mortality, calling for implementation of strategies to reduce gut microbiota disruption in this setting.

## Supplementary Information

Below is the link to the electronic supplementary material.

Supplementary file1 (DOCX 27 KB)

## Data Availability

Data are made available by the corresponding author upon reasonable request.
