# Supplementary material for: Clinical burden of Clostridioides difficile infection in infective endocarditis: a single-center experience
Source: Intern Emerg Med. 2025 Aug 11;20(8):2341–8. doi: 10.1007/s11739-025-04085-0 (PMC12672736; doi:10.1007/s11739-025-04085-0)
Supplement: Supplementary file 1 — Supplementary file1 (DOCX 27 KB) [file 11739_2025_4085_MOESM1_ESM.docx]

**Supplementary Table 1.**

Univariable analysis of clinical, biochemical and outcome variables comparing patients with and without diarrhea during antimicrobial treatment for infective endocarditis (n=370).

| **Parameter** | **Univariable analysis** | | |
| --- | --- | --- | --- |
|  | **Diarrhea during IE therapy** | | ***p-value*** |
|  | **Yes**  **(n = 50)** | **No**  **(n = 320)** |  |
| **General Characteristics** |  |  |  |
| **Age** | 65 [55 – 74.2] | 65 [52.2 – 73.7] | 0.674 |
| **Sex**  M  F | 25 (50)  25 (50) | 227 (70.9)  93 (29.1) | **0.003** |
| **Type of acquisition**  **Community-acquired**  **Healthcare-associated**  **Hospital-acquired** | 32 (64)  5 (10)  12 (24) | 238 (74)  28 (8)  36 (11) | 0.052 |
| **Vegetation size, mm** | 13.5 [7 – 18] | 13.5 [9 – 20] | 0.465 |
| **Type of valve**  Prosthetic biological valve  Prosthetic mechanical valve  Native valve  CIED lead  TAVI  Multisite location  Other | 9 (18)  8 (16)  20 (40)  6 (12)  2 (4)  3 (6)  3 (6) | 42 (13.1)  35 (10.9)  158 (49.3)  41 (12.8)  10 (3.1)  19 (5.9)  19 (5.9) | 0.850 |
| **Vegetation location**:  Aortic valve  Mitral valve  Multivalve/multisite involvement  CIED lead  Tricuspid valve  Other  Pulmonary valve | 14 (28)  18 (36)  10 (20)  4 (8)  2 (40)  1 (2)  1 (2) | 118 (36.8)  87 (27.1)  51 (15.9)  34 (10.6)  14 (4.3)  7 (2.1)  9 (2.8) | 0.810 |
| **Causative microorganism:**  Streptococcus spp.  Staphylococcus aureus  Coagulase-egative Staphylococci  Enterococcus spp.  Gram-negatives  Candida spp.  Corynebacterium spp.  Negative cultures | 4 (8)  14 (28)  5 (10)  14 (28)  2 (4)  1 (2)  2 (4)  8 (16) | 82 (25.6)  62 (19.3)  47 (14.6)  58 (18.1)  5 (1.5)  1 (0.3)  2 (0.6)  59 (18.4) | **0.014** |
| **Biochemical data** |  |  |  |
| C-reactive protein, mg/dL | 9.9 [6 – 19.1] | 7.2 [3.8 – 13.2] | **0.026** |
| Creatinine, mg/dL | 1.05 [0.7 – 1.7] | 1 [0.8 – 1.4] | 0.855 |
| NT-proBNP, pg/mL | 4108 [1635 – 6786] | 2508 [615 – 8565] | 0.163 |
| Troponin, ng/mL | 14.9 [0.09-63.32] | 8.1 [0.08-53.2] | 0.482 |
| D-dimers, ng/mL | 1218.5 [508.7 – 2599.5] | 949 [495 – 2192.5] | 0.348 |
| White blood cells, n/mcL | 11595 [7820 – 16172.5] | 10240 [7675 – 13475] | 0.254 |
| **Antimicrobials before blood cultures** | 12 (24) | 78 (24) | 0.954 |
| **Duration of antibiotic therapy, days** | 32.5 [21.7 – 42.2] | 29 [19 – 42] | 0.175 |
| **Comorbidities** |  |  |  |
| Chronic Heart Failure (prior to IE onset) | 17 (34) | 78 (24.3) | 0.147 |
| Ischemic Heart Disease | 13 (26) | 71 (22.1) | 0.550 |
| Chronic Obstructive Pulmonary Disease | 14 (28) | 70 (21.8) | 0.336 |
| Diabetes | 14 (28) | 69 (21.5) | 0.310 |
| Liver disease | 17 (34) | 41 (12.8) | **< 0.001** |
| Malignant neoplasia | 8 (16) | 49 (15.3) | 0.900 |
| Chronic kidney disease | 20 (40) | 108 (33.7) | 0.413 |
| Peripheral artery disease | 9 (18) | 47 (14.6) | 0.543 |
| **Charlson comorbidity index** | 4.5 [2 – 6.2] | 4 [2 – 6] | 0.173 |
| **Antimicrobial therapy:**  Amoxicillin-clavulanic acid  Ampicillin-sulbactam  Cefazolin  Ceftriaxone  Daptomycin  Teicoplanin  Vancomycin | 6 (12)  12 (24)  5 (10)  5 (10)  13 (26)  1 (2)  2 (4) | 52 (16.2)  70 (21.8)  15 (4.6)  43 (13.4)  87 (27.1)  14 (4.3)  6 (1.8) | 0.442  0.737  0.122  0.501  0.860  0.428  0.337 |
| **Cardiac Surgery**  Yes  No | 30 (60)  20 (40) | 195 (60.9)  125 (39.1) | 0.900 |
| **Length of hospitalization, days** | 33 [24.5 – 45.2] | 26 [16.2 – 39] | **0.008** |
| **In-hospital outcome:**  Survivors  Non-survivors | 39 (78)  11 (22) | 261 (81.5)  59 (18.4) | 0.550 |

Categorical variables are presented as number and percentage. Numerical variables are presented as median and IQR

CIED: cardiac implatable electronic devices; TAVI: transcatheter aortic valve implant;
